# Stability and trajectory tracking of four- wheel steering trackless auxiliary transport robot via PID control

**DOI:** 10.3389/frobt.2025.1617376

**Published:** 2025-06-25

**Authors:** Mingrui Hao, Yueqi Bi, Jie Ren, Lisen Ma, Jiaran Li, Sihai Zhao, Miao Wu

**Affiliations:** ^1^ Campus School of Mechanical and Electrical Engineering, China University of Mining and Technology-Beijing, Beijing, China; ^2^ China Coal Technology and Engineering Group Taiyuan Research Institute Co. Ltd., Taiyuan, China; ^3^ Industrial Artificial Intelligence Technology Research Center, Taiyuan Institute of Technology, Taiyuan, China

**Keywords:** trackless auxiliary transport robot, four wheel steering, trajectory tracking, robot maneuvering stability, PID control algorithm

## Abstract

In the complex working environment of underground coal mines, narrow road conditions and deviation in the driving path of autonomous trackless auxiliary transport robots can easily lead to collisions with walls or obstacles. This issue can be effectively solved by a four-wheel steering system, as it can reduce the turning radius of the robot at low speeds and improve its maneuverability at high speeds. Thus, a linear two-degree-of-freedom dynamics model of trackless auxiliary transport robot is established and the steady-state lateral critical speed of 16.6 km/h is obtained. Then a four wheel steering PID trajectory tracking strategy were constructed. Experiments on different steering modes at low and high speeds, which include stepped steering angles and circular path tracking, for the front-wheel steering mode and four-wheel steering mode of the robot are conducted under loaded conditions. The experimental results show that in the low-speed 10 km/h step steering angle input test, compared with the front-wheel steering mode, the turning radius of the robot is reduced by 32.2%, which ensures it easier to pass through narrow tunnels. Under the conditions of a 40 km/h high-speed step steering angle input test, the handling stability has been improved. The results of the circular trajectory tracking test show that at low speeds (10 km/h), the average radius error of the robot is 0.3%, while the radius error of the front-wheel steering robot reaches 2.12%. At high speeds (40 km/h), the average radius error is 2.4%, while the radius error of front-wheel steering mode is 8.74%. The robot maintains good track tracking ability, reducing the risk of collision with tunnel walls and improving robot operation safety.

## 1 Introduction

Mine auxiliary transportation system is one of crucial part of the entire mining production system, which affords the transporting personnel and materials in underground environments. The technical level and operational efficiency of the system directly influence both mine production rates and workers safety. Auxiliary transport vehicles which powered by electric motors and wheel-type vehicles is a primary mode of transportation. This type vehicle can provide flexibility and point-to-point transportation capabilities. However, the confined and poorly ventilated spaces within underground tunnels result in emissions from diesel engines and vehicle noise significantly impairing the physical health of underground workers. Electric wheel mining transport robots are increasingly preferred to enhance working environment quality, reduce noise levels, and boost safety for underground workers (Hao et al., 2020; Zhang et al., 2022; Wang et al., 2020). A notable challenge with electric wheel mine vehicles is their steering system, particularly in narrow underground tunnels where deviations or delays in turning can lead to accidents by colliding with tunnel walls or obstacles. Implementing a four wheel steering system ensures the vehicle maintains its yaw angle while accurately tracking the roll angle, and it effectively suppresses wheel spin through a longitudinal slip controller based on a four wheel independent steering mechanism under various conditions (Hu et al., 2015; Nasri et al., 2016; Tabbache et al., 2011; Zhao and Zhang, 2009). This system is designed to prevent wheel spin in all scenarios, including high-speed operation. The yaw angle control by the four wheel steering system ensures that the vehicle’s orientation remains optimal during traversal, which not only prevents excessive roll angle drift but also enhances stability and safety for underground workers. When operating at low speeds, the four wheel steering system allows for precise control over the vehicle’s direction, making it possible to navigate through narrow underground tunnels effectively (Cao and Qiao, 2017; Khan et al., 2019; Jeong and Yim, 2022; Wang et al., 2018). Additionally, in emergency scenarios where sudden turns or obstacle avoidance is required, the four wheel steering system provides enhanced maneuverability, enabling the vehicle to execute smooth and controlled turns while avoiding obstacles. This feature improves the vehicle’s operational stability at high speeds (Zhu et al., 2023; Zhang et al., 2023; Ahmadian et al., 2022).

Current researchers in vehicle and omni-wheeled robot four wheel steering systems focuses on the design of four wheel steering mechanisms, research of control algorithms for four wheel steering, and research of trajectory tracking algorithms for four wheel steering. The type of execution mechanism determines the control algorithms used. For example, Kosmidis et al. (2023) proposed a neural network and fuzzy control algorithm to control the four steers of an electro-motor vehicle using a neural network and fuzzy logic simultaneously, ensuring that even if one wheel fails, the vehicle remains on course. Li et al. (2018) developed a hydrodynamic torque-controlled four wheel steering model based on the principle of differential steering and used equilibrium technology to adapt different speed modes for steering differential control through simulation and experimental testing, showing consistency between calculated and measured vehicle turning radii. Yu et al. (2019) proposed a nonlinear three-step heading controller for four wheeled controlled steering vehicles to improve operate stability by following the reference yaw rate and lateral slip angle. Their nonlinear three-step heading controller controls the front and rear steering angles to track these reference values, ensuring effective tracking accuracy even when operating in highly nonlinear wheel regions. Song et al. (2017) developed a biased differential (OCS) and fully automatic tracking four wheel steering system for all-direction non-coupled flexible chassis vehicles (FC), with the turning wheels’ steering axes driven by electric brake in open or closed positions to overcome geometric characteristics issues inherent in over-mechanization, using a Whistone bridge for steering control. By monitoring changes in bridge resistors to track target and actual turning angles. Bae and Lee (2023) designed a compact four wheel independent steering mobile robot platform with adaptive four wheel steering control algorithms to enhance robot maneuverability so that the robot’s heading aligns with human operator intent. This platform uses front-wheel steering for normal operation and rear-wheel steering during additional turns to effectively reduce turning radii, as demonstrated through experiments confirming the precise steering performance of the robot under adaptive heading control algorithms. Haytham et al. (2014) designed an optimized PID controller for autonomous ground vehicles, achieving desired task paths and steering stability by minimizing lateral error between vehicle headings and target points. Meng et al. (2018) proposed a nonsmooth finite-time convergence controller based on an ideal electric vehicle steering tracking model to control the turning angles of the four wheels in electric vehicles, enhancing safety and maneuverability. The results indicate that non-smooth finite-time control methods are superior to sliding mode control methods for active four wheel steering systems of electric vehicles. Dai et al. (2024) focused on a four wheeled-driven and lateral-direction controlled autonomous chassis platform. A personalized path tracking control strategy based on reference vector field was proposed, utilizing distributed execution architecture for the four wheeled-driven Autonomous chassis and implementing lateral-direction control to enable multi-directional driving capabilities for the autonomous chassis. Özatay (2003) employed a fuzzy logic controller that integrates driver inputs for four wheel steering systems, ensuring zero body lateral deviation angle and rapid response of yaw rate. Lam et al. (2014) studied longitudinal anti-slip controllers for four wheel independent steering longitudinal wheel slippage, using wheel speed sensors to monitor tire traction and accelerometers to monitor vehicle acceleration conditions. Compared to traditional front and rear two-wheeler steering systems, numerical simulation results demonstrated the effectiveness of the proposed longitudinal wheel slip avoidance controller under various operating conditions. Tavolo et al. (2024) proposed a nonlinear model integrated with four-wheel steering, longitudinal tire force distribution, and direct yaw moment control method for predictive control (NMPC), which controls the vehicle’s path tracking under drifting conditions. The simulation and experimental test results show that the algorithm has good control effect on vehicle drift. Yu et al. (2023) established a nonlinear model predictive controller that can ensure that the actual sideslip angle and yaw rate can track the ideal values, and control the sideslip angle and ideal yaw rate by controlling the front and rear wheel angles. The simulation results in CarSim show that the proposed controller can reduce computational burden, improve the handling stability of active four-wheel vehicles, and effectively manipulate the vehicle.

The project research team designed a trackless auxiliary transport robot, which adopts the design scheme of steering drive axles, each steering drive axle is driven by a high-power flat wire motor, and the two wheels are steered by a planetary ball screw electric cylinder, and the steering mechanism of each axle is an Ackerman steering mechanism. An angle sensor is installed between the wheel steering kingpin and the axle housing to measure the rotation angle of each wheel and a wheel speedometer is installed on each wheel to obtain the driving speed of the trackless auxiliary transport robot. The inertial measurement unit is installed on the robot to obtain the heading angle, lateral deviation angle and lateral acceleration information of the robot. In this study, the trackless auxiliary transport robot is taken as the research object and a linear two-degree-of-freedom dynamic model of the robot is established, which ignores tire dynamics and nonlinear issues of the system. The frame and axle are rigidly connected to adapt to rough working environments, so the model ignores the influence of suspension. The robot adopts solid tires and the frame is rigidly connected to the wheel axle to adapt to rough working conditions. Therefore, this model ignores the nonlinear issues of tire dynamics and the system and simplifies it into a linear model without suspension. Then, the steady-state steering critical velocity is obtained according to the dynamic model. A four-wheel steering control strategy and a PID trajectory tracking control strategy are constructed, both of which use driving speed as the control input and steering angle as the output. The experiments of the steering angle step of the wheeled transport robot under the full-load condition of the wheeled transport robot at three speeds of 10 km/h, 16.6 km/h and 40 km/h are carried out. In addition, the circular path following experiment of the wheeled transport robot with a radius of 50 m at two speeds of 10 km/h and 40 km/h was also tested.

## 2 Dynamic model of four wheel steering for trackless auxiliary transport robot

### 2.1 Two degree freedom model of four wheel steering robot

**FIGURE 1:**
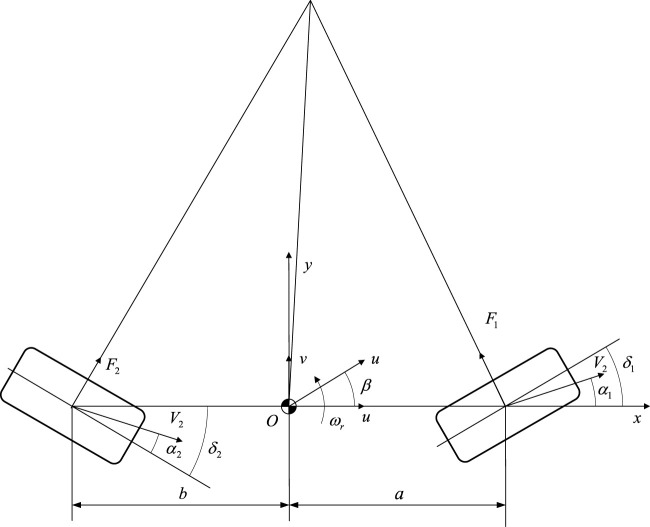
Two degree freedom model of four wheel steering trackless auxiliary transport robot.

The two degree freedom model of four wheel steering trackless auxiliary transport robot is shown in Figure 1. Where a and b are the distances from the center of mass of the trackless auxiliary transport robot to the front and rear axles, respectively. The u and v are the longitudinal and lateral velocities of the centroid of the robot respectively. $\omega_{r}$ represents the yaw angular velocity and $\alpha_{1}$ , $\alpha_{2}$ represent slip angle of the front and rear wheels of the robot respectively. β represents slip angle of the robot mass center. $\delta_{1}$ and $\delta_{2}$ represent steering angle of the front and rear wheels of the robot respectively.

The lateral acceleration at the center of mass of the trackless auxiliary transport robot is Equation 1.

$$a_{y}=\dot{v}+u\omega_{r}$$ (1)

The lateral offset angles of the front and rear wheels of the trackless auxiliary transport robot are given by Equations 2, 3 respectively.

$$\alpha_{1}=\beta +\frac{a\omega_{r}}{u}-\delta_{1}$$ (2)

$$\alpha_{2}=\beta -\frac{b\omega_{r}}{u}-\delta_{2}$$ (3)

The lateral force $F_{y1}$ of the front wheels of the trackless auxiliary transport robot are by Equation 4. In Equation 4 $K_{\alpha 1}$ represent the lateral stiffness of the front wheels.

$$F_{y1}=K_{\alpha 1}\alpha_{1}$$ (4)

The lateral force $F_{y2}$ of the rear wheels of the trackless auxiliary transport robot are by Equation 5. The $K_{\alpha 2}$ means the lateral stiffness of the rear wheels.

$$F_{y2}=K_{\alpha 2}\alpha_{2}$$ (5)

According to Newton’s laws, the differential equations for a two-degree -freedom model of four wheel steering trackless auxiliary transport robot is shown as Equations 6, 7.

$$m\left(\dot{v}+u\omega_{r}\right)=F_{y1}+F_{y2}$$ (6)

$$I_{z}{\dot{\omega }}_{r}=aF_{y1}-bF_{y2}$$ (7)

In Equation 7, m represents the mass and $I_{z}$ represents the moment of inertia of a trackless auxiliary transport robot, respectively.

Merging Equations 2, 3 into Equations 6, 7. Then Equations 8, 9 can be get as follows (Ackermann et al., 1993).

$$m\left(\dot{v}+u\omega_{r}\right)=K_{\alpha 1}\left(\beta +\frac{a\omega_{r}}{u}-\delta_{1}\right)+K_{\alpha 2}\left(\beta -\frac{b\omega_{r}}{u}-\delta_{2}\right)$$ (8)

$$I_{z}{\dot{\omega }}_{r}=aK_{\alpha 1}\left(\beta +\frac{a\omega_{r}}{u}-\delta_{1}\right)-bK_{\alpha 2}\left(\beta -\frac{b\omega_{r}}{u}-\delta_{2}\right)$$ (9)

Arranging Equations 8, 9 into a matrix equation as form as Equation 10.

$$\left[\begin{array}{l} \dot{\beta }\\ {\dot{\omega }}_{r} \end{array}\right]=\left[\begin{array}{cc} a_{11} & a_{12}\\ a_{21} & a_{22} \end{array}\right]\left[\begin{array}{l} \beta \\ \omega_{r} \end{array}\right]+\left[\begin{array}{cc} b_{11} & b_{12}\\ b_{21} & b_{22} \end{array}\right]\left[\begin{array}{l} \delta_{1}\\ \delta_{2} \end{array}\right]$$ (10)

In matrix Equation 10, each element of the matrix block is shown in follows.

$$a_{11}=\frac{K_{\alpha 1}+K_{\alpha 2}}{mu};\;a_{12}=\frac{aK_{\alpha 1}-bK_{\alpha 2}-mu^{2}}{mu^{2}};\;a_{21}=\frac{aK_{\alpha 1}-bK_{\alpha 2}}{I_{z}};$$

$$a_{22}=\frac{a^{2}K_{\alpha 1}+b^{2}K_{\alpha 2}}{I_{z}u};\;b_{11}=-\frac{K_{\alpha 1}}{mu};\;b_{21}=-\frac{aK_{\alpha 1}}{I_{z}};\;b_{12}=-\frac{K_{\alpha 2}}{mu};\;b_{22}=\frac{bK_{\alpha 1}}{I_{z}}$$

Equation 11 is obtained by performing the Laplace transform in Equation 10.

$$\left[\begin{array}{cc} a_{11} & a_{12}\\ a_{21} & a_{22} \end{array}\right]\left[\begin{array}{l} \beta \left(s\right)\\ \omega_{r}\left(s\right) \end{array}\right]=\left[\begin{array}{cc} b_{11} & b_{12}\\ b_{21} & b_{22} \end{array}\right]\left[\begin{array}{l} \delta_{1}\left(s\right)\\ \delta_{2}\left(s\right) \end{array}\right]$$ (11)

Using Cramer’s rule (Cramer, 1750) the transfer function of the centroid declination angle of the trackless auxiliary transport robot is Equation 12.

Gβs=b11s+ab2112−a22b11s2−a11+a22s+a11a22−a12a21δ1s+b12s+ab2212−a22b12s2−a11+a22s+a11a22−a12a21δ2s (12)

The yaw rate transfer function of the trackless auxiliary transport robot is shown in Equation 13.

Gωrs=b21s+ab1121−a11b21s2−a11+a22s+a11a22−a12a21δ1s+b22s+ab1221−a11b22s2−a11+a22s+a11a22−a12a21δ2s (13)

The four wheel steering system uses proportional control of the front wheel steering angle, with the control objective being a zero lateral deviation angle in steady-state operation, and the rear wheel steering angle is given by Equation 14.

$$\delta_{2}\left(s\right)=G_{21}\delta_{1}\left(s\right)$$ (14)

In Equation 14, $G_{21}$ is the ratio of the steering angles of the front and rear wheels.

Substituting Equation 14 into Equation 13, the transfer function of the yaw rate of the trackless auxiliary transport robot is shown in Equation 15.

$$\frac{G_{\omega_{r}}\left(s\right)}{\delta_{1}\left(s\right)}=\frac{\left(b_{21}+G_{21}b_{22}\right)s+a_{21}\left(b_{11}+G_{21}b_{12}\right)-a_{11}\left(b_{21}+G_{21}b_{22}\right)}{s^{2}-\left(a_{11}+a_{22}\right)s+a_{11}a_{22}-a_{12}a_{21}}$$ (15)

The transfer function of the centroid declination angle of the trackless auxiliary transport robot is Equation 16.

$$\frac{G_{\beta }\left(s\right)}{\delta_{1}\left(s\right)}=\frac{\left(b_{11}+G_{21}b_{12}\right)s+a_{12}\left(b_{21}+G_{21}b_{22}\right)-a_{22}\left(b_{11}+G_{21}b_{12}\right)}{s^{2}-\left(a_{11}+a_{22}\right)s+a_{11}a_{22}-a_{12}a_{21}}$$ (16)

When $G_{21}=0$ , the transfer functions of centroid lateral deviation angle and yaw rate of the front-wheel steering trackless auxiliary transport robot can be obtained.

When the trackless auxiliary transport robot travels stably, the yaw rate is a constant value and the differential term is zero.

Equation 17 can be obtained from Equation 11.

$$\left[\begin{array}{l} \beta \\ \omega_{r} \end{array}\right]=-{\left[\begin{array}{cc} a_{11} & a_{12}\\ a_{21} & a_{22} \end{array}\right]}^{-1}\left[\begin{array}{l} b_{11}+b_{12}G_{21}\\ b_{21}+b_{22}G_{21} \end{array}\right]\delta_{1}$$ (17)

According to Equations 15–17, the steady-state center of mass lateral deviation angle can be obtained as Equation 18.

$$\beta =\frac{\left|\begin{array}{cc} b_{11}+b_{12}G_{21} & a_{12}\\ b_{21}+b_{22}G_{21} & a_{22} \end{array}\right|}{\left|\begin{array}{cc} a_{11} & a_{12}\\ a_{21} & a_{22} \end{array}\right|}\delta_{1}$$ (18)

To ensure that the steady-state lateral deviation angle is always zero, there should be Equation 19.

$$\left|\begin{array}{cc} b_{11}+b_{12}G_{21} & a_{12}\\ b_{21}+b_{22}G_{21} & a_{22} \end{array}\right|=0$$ (19)

At this point, the ratio of the front and rear wheel angles $G_{21}$ can be represented by Equation 20 (Ulsoy et al., 2012).

$$G_{21}=\frac{a_{12}b_{21}-a_{22}b_{11}}{a_{22}b_{12}-a_{12}b_{22}}=\frac{mu^{2}aK_{\alpha 1}+bLK_{\alpha 1}K_{\alpha 2}}{mu^{2}bK_{\alpha 2}-aLK_{\alpha 1}K_{\alpha 2}}$$ (20)

If the front and rear wheel steering angle ratios are set according to Equations 15–20, it can ensure that the center of mass lateral deviation angle of the car during steady-state driving is zero, which varies with the speed of the robot’s movement. The critical velocity $u_{0}$ can be calculated by Equation 21.

$$u_{0}=\sqrt{-\frac{bLK_{\alpha 2}}{ma}}$$ (21)

At the critical speed $u_{0}$ , the ratio $G_{21}$ of the steering angles of the front and rear wheels is about zero and the four wheel steering system is equivalent to the front wheel steering system. The steering angle ratio $G_{21}$ of the front and rear wheels of the four-wheel steering system is set according to Equation 21. Table 1 shows the parameters of the trackless auxiliary transport robot used in Equation 21.

**TABLE 1:** Parameters of trackless auxiliary transport robots.

Symbol	Expression	Value
*M*	Mass of robot	10,000 kg
*K*_*α1*_	Lateral stiffness of the front wheels	−85,000 N/rad
*a*	Centroid to front axle distance	1.25 m
*Iz*	Rotational inertia about the Z-axis	25,392 kg m^2^
*K*_*α2*_	Lateral stiffness of the rear wheels	−85,000 N/rad
*B*	Centroid to rear axle distance	1.25 m

**FIGURE 2:**
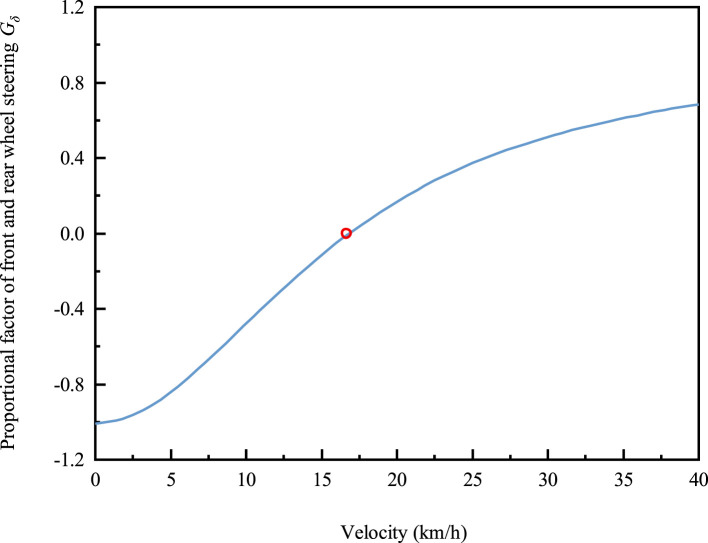
The ratio of the steering angles of the front and rear wheels as a function of vehicle speed.

The ratio of the steering angle of the front and rear wheels is calculated as shown in Figure 2, and the critical speed $u_{0}$ is 16.6 km/h. When the vehicle speed is less than $u_{0}$ , that is low speed condition, the ratio of the steering angle of the front and rear wheels $G_{21}$ is negative, and the steering angle direction of the front and rear wheels is opposite. When the vehicle speed is greater than $u_{0}$ , that is the high speed condition, the ratio of the steering angle of the front and rear wheels $G_{21}$ is positive, the steering angle directions of the front and rear wheels are the same.

### 2.2 PID trajectory tracking controller design

The PID controller is a typical dynamic output feedback controller and its control law is shown in Equation 22.

$$\delta_{1}\left(t\right)=K_{p}e\left(t\right)+K_{i}\int_{0}^{t}e\left(\tau \right)d\tau +K_{d}\dot{e}\left(t\right)$$ (22)

In Equation 22, $K_{p}$ , $K_{i}$ , and $K_{d}$ are the proportional coefficient, integral coefficient, and differential coefficient of the error, respectively. The PID controller is suitable for single input single output systems and it is difficult to apply it to the control system of front and rear wheel steering trackless auxiliary transport robots. Therefore, the input strategy for rear wheel steering angle control is selected according to Equation 14 and the system measurement input is selected as measurable yaw rate which is easy to get this data from IMU. In addition, turning radius of the actual path can be calculated by yaw rate and turning radius error between planned path and actual path is equal to lateral displacement in the robot coordinate system. Thus, the single input single output system model is designed as Equation 23.

$$\left\{\begin{array}{l} \dot{x}=Ax+Bu\\ y=Cx \end{array}\right.$$ (23)

In Equation 23, each parameter is shown in Equation 24.

$$\begin{array}{ll} x & =\left[\begin{array}{l} v\\ \omega_{r} \end{array}\right],y=\omega_{r},u=\delta_{1},B=\left[\begin{array}{l} -\frac{K_{\alpha 1}+G_{21}K_{\alpha 2}}{m}\\ \frac{G_{21}K_{\alpha 2}b-K_{\alpha 1}a}{I_{z}} \end{array}\right],C=\left[\begin{array}{cc} 0 & 1 \end{array}\right],\\ & \;A=\left[\begin{array}{cc} \frac{K_{\alpha 1}+K_{\alpha 2}}{mu} & \frac{K_{\alpha 1}a-K_{\alpha 2}b_{r}}{mu}-u\\ \frac{K_{\alpha 1}a-K_{\alpha 2}b}{I_{z}u} & \frac{K_{\alpha 1}a^{2}+K_{\alpha 2}b^{2}}{I_{z}u} \end{array}\right] \end{array}$$ (24)

The system Equation 23 is a single-input single-output system, and a PID controller can be designed for the model. Equation 25 is the method for calculating the yaw rate $\omega_{ref}$ , $R_{0}$ is the turning radius of the planned path.

$$\omega_{ref}=\frac{u}{R_{0}}$$ (25)

Then the yaw rate tracking error of the PID controller Equation 22 is Equation 26.

$$e\left(t\right)=\omega_{ref}\left(t\right)-\omega_{r}\left(t\right)=\frac{u}{R_{0}}-\omega_{r}\left(t\right)$$ (26)

In this research, the parameters $K_{p}$ , $K_{i}$ , and $K_{d}$ of the PID controllers for the four-wheel steering mode and front wheel steering mode of the vehicle were set to 1.0, 0.95, and 0.012, respectively.

## 3 Design and testing method of four-wheel steering system

### 3.1 Design of four wheel steering system for trackless auxiliary transport robot

**FIGURE 3:**
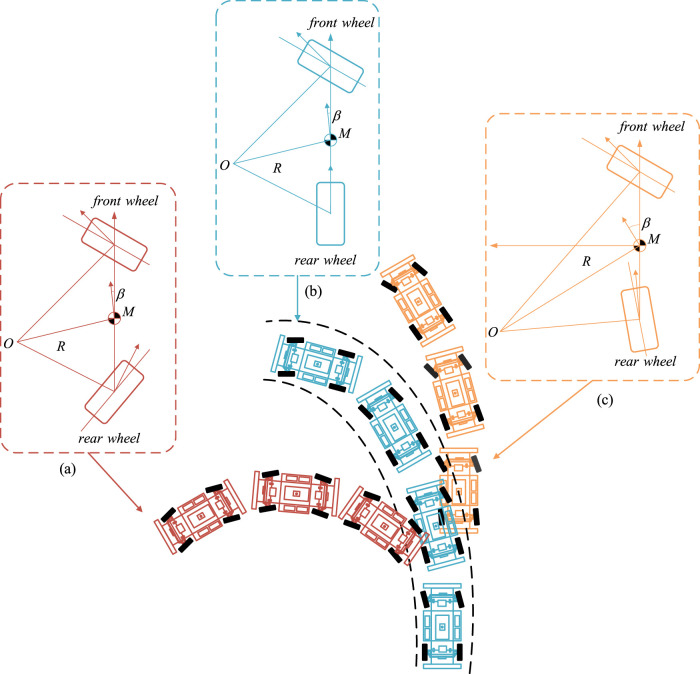
Steering motion of four wheel steering trackless auxiliary transport robot. **(a)** Over steering. **(b)** Neutral steering. **(c)** Under steering.

The basic principle of the four-wheel steering system is to control the angle input of the front and rear wheels by using the driving speed of the trackless auxiliary transport robot and the steering wheel angle signal input from the automatic driving system, so as to improve the trafficability, maneuverability and stability of the robot. As shown in Figure 3, when the robot travels at low speed, to reduce the turning radius, the robot usually the rear wheel steering direction is opposite to the front wheel, which is defined as “over steering.” In over-steering state, the turning radius of robot is reduced but the yaw rate and lateral velocity is increased that decrease the stability of the robot; During high-speed steering, to improve the stability and speed up the lateral response speed of the robot, the rear wheel will produce the steering angle in the same direction as the front wheel, which is named “under steering,” the robot in state of understeer, although the stability and lateral speed response of the robot are improved in this state, the turning radius of the robot increases.

**FIGURE 4:**
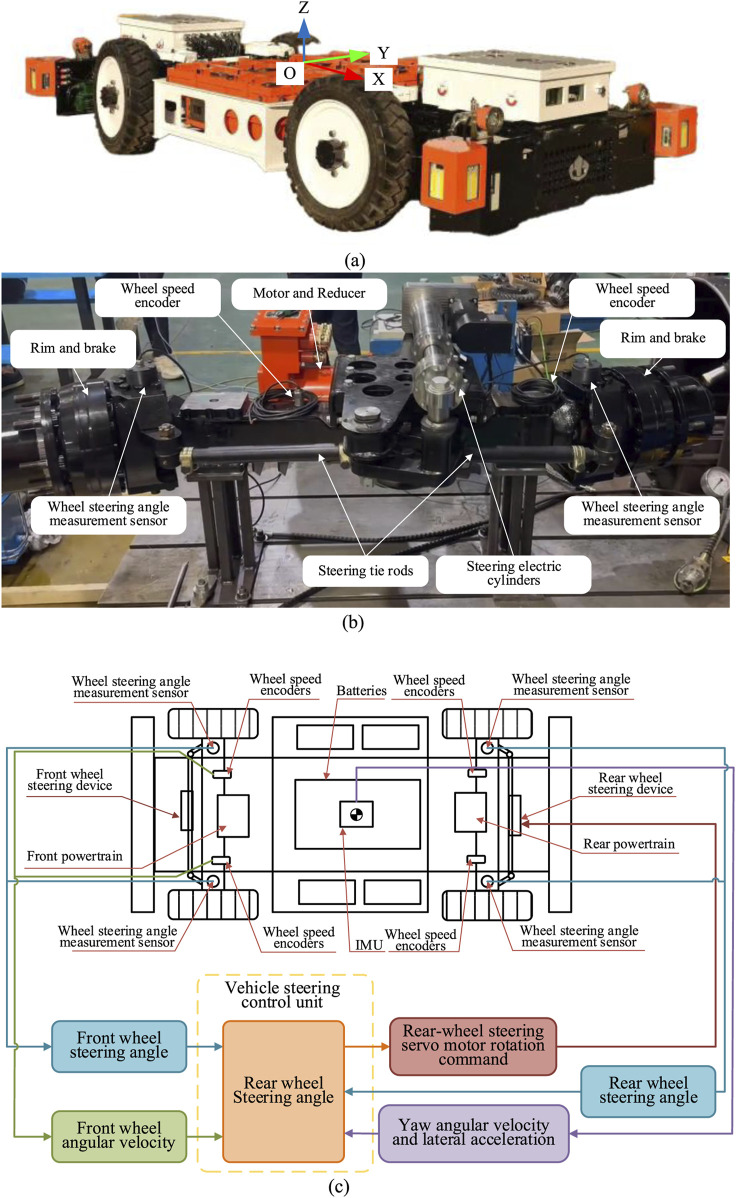
Principle of four wheel steering control system. **(a)** Trackless auxiliary transport robot condinate system. **(b)** Steering electric drive axle. **(c)** Four wheel steering control system.

The control of the rear wheel rotation angle of the four wheel steering system is not only related to the robot speed, but also related to the movement state of the robot such as the front wheel rotation angle, yaw angle velocity and its control strategy is realized by using the electronic control system. As shown in Figure 4. Figure 4a shows the robot structure and coordinate system. The system original point *O* is at the robot mass center and the X direction is the forward direction of the robot. The robot adopts the steering electric drive axle as shown in Figure 4b. The wheeled odometer is installed on the steering drive axle half shaft for measuring the forward speed of the robot. The steering angle sensor is installed on the steering drive axle kingpin for measuring the steering angle of the robot. The steering mechanism drive device is a servo-electric cylinder. The wheel steering is realized through the telescopic push-pull steering transmission mechanism of the servo-electric cylinder. The four wheels steering control system is shown in Figure 4c, firstly, the front wheel steering signal and the robot motion state are sent to the steering control unit. Then, the steering angle of rear wheels is analyzed and calculated according Equation 27. $G_{21}\left(u\right)$ represents the ratio of front and rear wheel steering, which is related to the robot’s driving speed.

$$\delta_{2}=G_{21}\left(u\right)\cdot \delta_{1}$$ (27)

Secondly, the front and rear wheel steering servo axes are controlled to perform the corresponding steering action and the front and rear wheels are driven to deflect by the steering transmission mechanism. At the same time, the steering control unit monitors the movement state of and adjusts the rear wheel rotation angle of the robot in real time, which realizes the four wheel steering of the robot ultimately.

### 3.2 Experiment on four wheel steering system of trackless auxiliary transport robot

An angle sensor is installed between the wheel steering pin and the axle housing to measure the angle of each wheel, which is type of a single coil absolute value magnetoresistance angle sensor, with a sampling frequency of 50 Hz. One wheel-speed sensor per wheel is installed on each wheel to obtain the driving speed of the robot. The wheel encoder adopts Hall type gear speed sensor of which 50 Hz sampling frequency and 1,024 pulses/rev. The robot is equipped with an inertial measurement unit to obtain the heading angle, lateral deviation angle and lateral acceleration information of the robot. The IMU can get the acceleration values of three directions x-y-z and rate values of yaw, pitch and roll. The output frequency of IMU is 400 Hz. RTK positioning system is installed to measure the trajectory and lateral displacement of the robot. The sampling frequency is 50 Hz.

(1) Step angle input working condition test: The vehicle speed is set to three conditions of 10 km/h, 16.6 km/h, and 40 km/h. And the steering mode of the robot is set in front wheel steering mode and four-wheel steering mode. The front wheel angle input of each mode is 0.17453 rad. The testing time is 10 s. In the four-wheel steering mode of the robot, the rear wheel angle is automatically output based on the ratio $G_{21}\left(u\right)$ . Test result data post back frequency is 50 Hz.
(2) Circular path trajectory tracking: the vehicle speeds were set to 10 km/h and 40 km/h. And the steering mode of the robot is also set in front wheel steering mode and four-wheel steering mode. The target trajectory of the robot is set to a circular path with a radius of 50 m. The front wheel steering angles of each steering mode are both sine function inputs of which amplitude of the front wheel steering angle is 0.0105 rad, the frequency of low speed 10 km/h is 1 Hz and the frequency of high speed 40 km/h is 1.25 Hz. Test result data post back frequency is 50 Hz.

## 4 Test results and discussion

### 4.1 Test results of step angle input conditions

**FIGURE 5:**
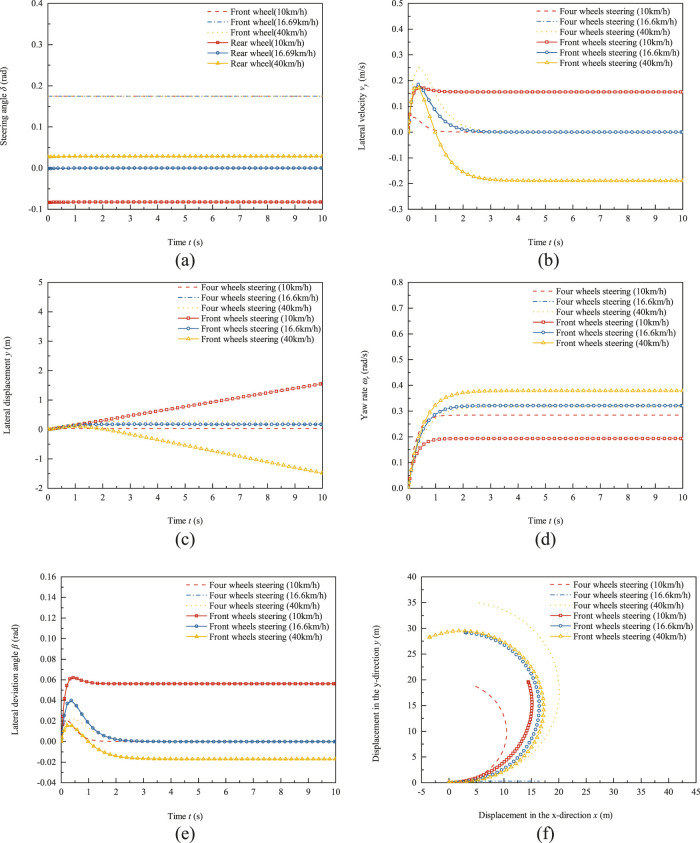
Test results of front wheel steering and four-wheel steering of trackless auxiliary transport robot under condition of front wheel steering angle step input. **(a)** Front and rear wheel steering angle. **(b)** Lateral velocity response. **(c)** Lateral displacement response. **(d)** Yaw rate response. **(e)** Lateral deviation angle response. **(f)** Driving trajectory.

The vehicle speed is set to three working conditions, which is 10 km/h, 16.6 km/h and 40 km/h, respectively. In Figure 5, the lateral displacement and lateral velocity are both positive values in the Y direction and negative values in the opposite direction of the robot coordinate system. The yaw rate and lateral angle are both positive values in the counterclockwise direction and negative values in the clockwise direction of the robot coordinate system Z-axis. The front wheel angle input of both the two wheel steering trackless auxiliary transport robot and the four-wheel steering mode is 0.175 rad. The front and rear wheel angle inputs of the four-wheel steering trackless auxiliary transport robot are shown in Figure 5a. At 10 km/h, the rear wheel steering angle is −0.0834 rad, the front and rear wheel steering angles are opposite and the ratio of the rear wheel steering angle to the front wheel steering angle. When the vehicle speed is 16.6 km/h, the rear wheel steering angle is 0 rad, and the ratio of the rear wheel steering angle to the front wheel steering angle, at which point four-wheel steering mode degenerates into front wheel steering mode. When the vehicle speed is 40 km/h, the rear wheel steering angle is 0.119 rad and the ratio of the rear wheel steering angle to the front wheel steering angle is the same for the front and rear wheels.

The comparison results of lateral velocity response between the front wheel steering mode and the four-wheel steering mode of the robot under the condition of front wheel steering angle step input are shown in Figure 5b. In Figure 5b, negative lateral velocity indicate offset in the negative direction relative to the robot coordinate system, while positive lateral velocity indicate offset in the positive direction relative to the robot coordinate system. On the other hand, negative values indicate that the robot is in an oversteer state, while positive values indicate that the robot is in an understeer state. At speeds of 10 km/h, 16.6 km/h, and 40 km/h, the lateral velocity of the four-wheel steering trackless auxiliary transport robot during steady-state motion is 0 m/s. The lateral velocity during steady-state motion of the front wheel steering mode at 10 km/h is 0.15 m/s and the lateral velocity during steady-state motion at 16.6 km/h is 0 m/s, which is the same as the four-wheel steering mode degraded to front wheel steering mode. The lateral velocity during steady-state motion of the 40 km/h trackless auxiliary transport robot is −0.189 m/s. The proportional coefficient control of front and rear wheel steering with zero lateral deviation angle as the target can achieve zero lateral displacement of the four wheel steering mode, especially under high-speed conditions, when the front and rear wheels turn in the same direction, it increases the tendency of the trackless auxiliary transport robot to understeer, and its lateral displacement is much smaller than that of the front wheel steering trackless auxiliary transport robot.

The lateral displacement curve of the robot under angular step conditions is shown in Figure 5c. The lateral displacement in Figure 5b, negative values represent offset in the negative direction relative to the robot coordinate system, while positive values represent offset in the positive direction relative to the robot coordinate system. At the same time, negative values represent that the robot is in an oversteer state, while positive values represent that the robot is in an understeer state. Due to the use of zero lateral deviation angle proportional coefficient to control the front and rear wheel angles of the four wheel steering trackless auxiliary transport robot, under three robot speed conditions of 10 km/h, 16.6 km/h, and 40 km/h, the lateral displacements of four wheel steering mode are 0.0332 m, 0.0174 m, and 0.282 m at the end time of tests, respectively. The lateral displacement of the front wheel steering mode at 10 km/h is 1.556 m. The lateral displacement at 16.6 km/h is 0.0174 m. At this point, the lateral displacement of the four wheels steering mode of robot that has degenerated into front wheel steering mode. At a high speed of 40 km/h, the lateral displacement of the robot with front wheel steering mode is −1.487 m at the end of tests. Compared to front wheel steering mode, the absolute lateral displacement of four wheels steering is 18.96% at a low speed of 10 km/h, while at a speed of 40 km/h is 2.056%.

Figure 5d shows the yaw rate response of the front wheel steering angle step input test of the robot. When the vehicle speed is 10 km/h, the steady-state yaw rate of four wheels steering mode is 0.284 rad/s and the steady-state yaw rate of front wheel steering mode is 0.193 rad/s. The steady-state yaw rate of four wheels steering mode is 1.47 times that of front wheel steering mode. When the vehicle speed is 16.6 km/h, four wheels steering mode degenerates into front wheel steering mode and its steady-state yaw rate is the same as front wheel steering mode, both at 0.321 rad/s. When the vehicle speed is 40 km/h, the steady-state yaw rate of four wheels steering mode is 0.316 rad/s and the steady-state yaw rate of front wheel steering mode is 0.378 rad/s. At high speeds, the steady-state yaw rate of four wheels steering mode is 83.6% of that of front wheel steering mode. Compared to front wheel steering mode, the steady-state yaw rate of four wheels steering mode is reduced by 16.5% under high-speed conditions. In addition, by observing the time for the yaw rate to reach steady state in Figure 5d, it can be found that when the vehicle speed is 10 km/h, the time for the yaw rate to reach steady state of the four wheels steering mode is 1.2 s but the time value of front wheel steering mode is 1.0 s. The reason for the longer stability time is that robots with four-wheel steering mode have a smaller turning radius and yaw rate compared to front wheel steering. When the vehicle speed is 40 km/h, the time for the four wheels steering mode yaw rate to reach steady state is just 2.2 s. However, that value of the front wheel steering mode is 2.8 s. Four wheels steering increases the turning radius and reduces yaw rate compared to front wheel steering, resulting in shorter stability time. This indicates that compared to front wheel steering mode, four wheels steering mode can quickly bring the robot into a stable state at high speeds, reduce the yaw rate of the robot and improve the safety of high-speed driving.

Figure 5e shows the response curve of the lateral deviation angle of the robot with a step input of the front wheel steering angle. When the vehicle speed is 10 km/h, the peak lateral deviation angle of four wheels steering mode is 0.0208 rad and the time to reach the peak is 0.205 s. The steady-state lateral deviation angle is 0 rad and the time to enter the steady state is 1.33 s. The peak lateral deviation angle of the front wheel steering mode is 0.0621 rad and the time to reach the peak is 0.436 s. The steady-state lateral deviation angle is 0.0561 rad and the time to enter the steady state is 1.52 s. At a speed of 16.6 km/h, the four wheel steering and rear wheel steering angles are zero, degraded to front wheel steering mode. At this time, the steady-state lateral deviation angle of four wheels steering mode and front wheel steering mode is, with a peak lateral angle of 0.0416 rad. The time to reach the peak is 0.371 s, the steady-state lateral deviation angle is 0 rad, and the time to enter steady state is 2.36 s. When the vehicle speed is 40 km/h, the four wheels steering mode lateral deviation angle reaches its peak at 0.0386 rad at 0.0226 s, enters steady state at 2.76 s, and the lateral deviation angle entering steady state is 0 rad. The front wheel steering mode lateral deviation angle enters steady state at the 3.36 s position, and the lateral deviation angle entering steady state is −0.018 rad. Through comparison, it was found that the faster the driving speed of the trackless auxiliary transport robot, the longer the time it takes for the lateral deviation angle to enter steady state. In addition, using a zero lateral deviation angle proportional coefficient to control the front and rear wheel angles of the four wheels steering trackless auxiliary transport robot, its steady state lateral deviation angle and response time are smaller than those of the front wheel steering mode, especially at a high speed of 40 km/h.

As shown in Figure 5f, the driving trajectory of the robot with angular step input. When the vehicle speed is at a low speed of 10 km/h, the turning radius of four wheels steering mode is 10.25 m, and the turning radius of front wheel steering mode is 15.12 m. Compared with front wheel steering mode, the turning radius of four wheels steering mode is reduced by 32.2%. When the vehicle speed is 16.6 km/h, the ratio coefficient of the rear wheel to the front wheel steering angle for four wheels steering mode is 0, that is, the rear wheel steering angle is 0 rad and four wheels steering mode degenerates into front wheel steering mode. Therefore, the trajectories of four wheels steering mode and front wheel steering mode coincide, with a turning radius of 16.41 m. When the robot is traveling at a high speed of 40 km/h, the turning radius of four wheels steering mode is 19.8 m, and the turning radius of front wheel steering mode is 16.9 m. The turning radius of four wheels steering mode is 17.4% greater than that of front wheel steering mode. Comparing the analysis results of Figure 5f, it can be seen that four wheel steering mode can significantly reduce the turning radius of the trackless auxiliary transport robot at low speeds, and improve the trafficability of the trackless auxiliary transport robot in narrow tunnel environments.

### 4.2 Trajectory tracking test results

**FIGURE 6:**
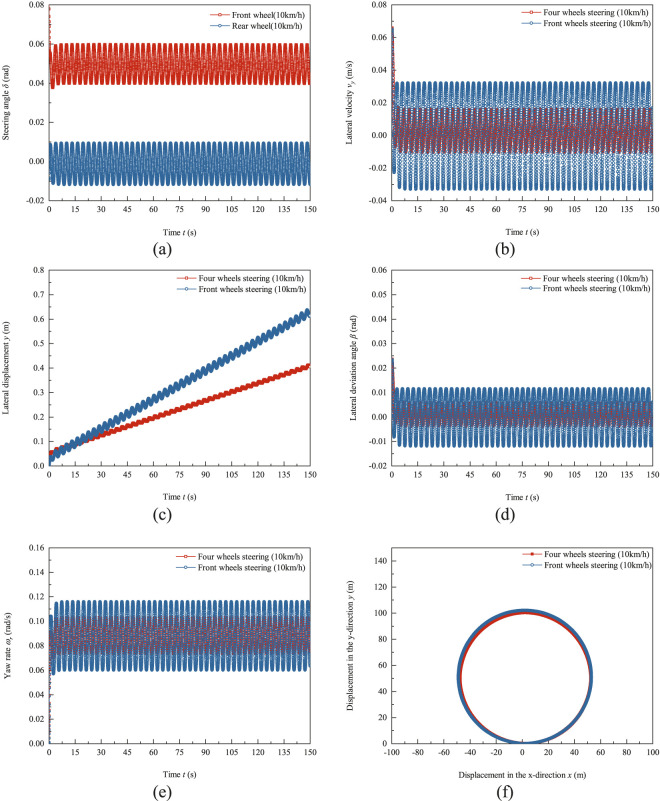
Test results of trajectory tracking of trackless auxiliary transport robot using PID control algorithm at a speed of 10 km/h **(a)** Front and rear wheel steering angle. **(b)** Lateral velocity response. **(c)** Lateral displacement response. **(d)** Lateral deviation angle response. **(e)** Yaw rate response. **(f)** Driving trajectory.

The trajectory tracking test results of the robot using PID control algorithm at a speed of 10 km/h are shown in Figure 6. The testing duration is 150 s, which is the horizons of Figure 6 from (a) to (e). In Figure 6, the lateral displacement and lateral velocity are both positive values in the Y direction and negative values in the opposite direction of the robot coordinate system. The yaw rate and lateral angle are both positive values in the counterclockwise direction and negative values in the clockwise direction of the robot coordinate system Z-axis. Figure 6a shows the input curve of the front and rear wheel angles. At a speed of 10 km/h, the ratio of the rear wheel to the front wheel steering of the robot is less than zero and the front and rear wheels turn in opposite directions. The front wheel angle is greater than zero, and the rear wheel angle is less than or equal to zero. The amplitude of the front wheel steering angle is 0.0105 rad, and the amplitude of the rear wheel steering angle is 0.01 rad. The average ratio $G_{21}$ of front and rear wheel angles is −0.919. Figure 6b shows the lateral velocity response curve, with a lateral velocity response amplitude of 0.01315 m/s and a deviation of 0.00284 m/s for four wheels steering. The lateral velocity response amplitude of the front wheel steering mode is 0.0325 m/s and the absolute offset is −0.0043 m/s. The lateral velocity response amplitude of four wheels steering is 40.46% of that of front wheel steering mode, and the absolute offset value is 66.05% of that of front wheel steering mode. Figure 6c shows the lateral displacement response curve. The curve indicates that the robot’s deviation from the 50 m circular radius. Positive deviation indicates that the robot is in an oversteer state and the actual turning radius is greater than the set radius by 50 m. The slope of the lateral displacement of the front wheel steering mode is significantly greater than that of the four wheels steering mode. At the test termination time t = 150 s, the lateral displacement of the front wheel steering mode reaches 0.62 m. The lateral displacement of four wheels steering mode is 0.4 m. The lateral deviation angle response curve is shown in Figure 6d. The amplitude of the lateral deviation angle of the four wheels steering trackless auxiliary transport robot is 0.00183 rad and the offset is 0.000026 rad. The amplitude of the front wheel steering lateral deviation angle is 0.00293 rad and the offset is −0.000038 rad. The amplitude of the four wheels steering lateral deviation angle is 62.45% of the amplitude of the front wheel steering lateral deviation angle and the absolute offset value is 68.42% of the front wheel steering.

Figure 6e shows the response curve of the yaw rate. The amplitude of the yaw rate for four wheels steering is 0.0145 rad/s, and the offset is 0.0885 rad/s. The amplitude of the yaw rate for front wheel steering mode is 0.0274 rad/s and the offset is 0.088 rad/s. Figure 6f shows the driving trajectory of the trackless auxiliary transport robot, with an average radius of 50.15 m and a radius error of 0.3% for the four wheels steering mode trajectory. The average radius of the front wheel steering mode trajectory is 51.06 m, with an average radius error of 2.12%. At a speed of 10 km/h, the four wheels steering mode trajectory tracking ability is superior to the front wheel steering.

**FIGURE 7:**
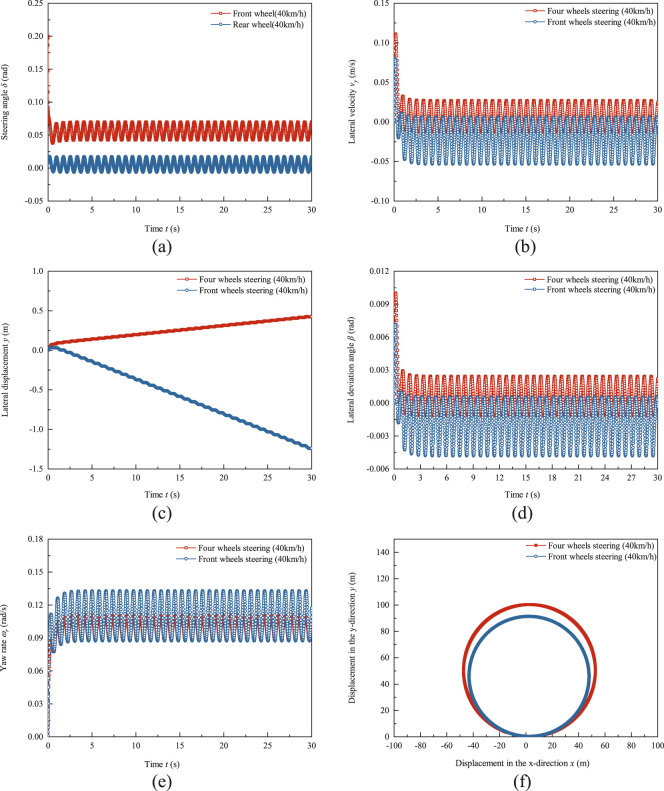
Test results of trajectory tracking of trackless auxiliary transport robot using PID control algorithm at a speed of 40 km/h. **(a)** Front and rear wheel steering angle. **(b)** Lateral velocity response. **(c)** Lateral displacement response. **(d)** Lateral deviation angle response. **(e)** Yaw rate response. **(f)** Driving trajectory.

The trajectory tracking test results of the robot using PID control algorithm at a speed of 40 km/h are shown in Figure 7. The testing duration is 30 s, which is the horizons of Figures 7a–e. The same axis specification is used for signed values in Figure 7 as in Figure 6. Figure 7a shows the input of front and rear wheel angles. Under high-speed conditions, the steering ratio of the rear wheel to the front wheel of the trackless auxiliary transport robot is greater than zero, and the front and rear wheels turn in the same direction. Therefore, both the front and rear wheel angles are greater than zero. The amplitude of the front wheel steering angle is 0.0137 rad and the offset is 0.056 rad, while the amplitude of the rear wheel steering angle is 0.0116 rad and the offset is 0.00519 rad. The average ratio $G_{21}$ of front and rear wheel angles is 5.62. Figure 7b shows the lateral velocity response curve, with a lateral velocity response amplitude of 0.0199 m/s and a deviation of 0.0071 m/s for four-wheel steering mode. The lateral velocity response amplitude of the front wheel steering mode is 0.0296 m/s, and the offset is −0.024 m/s. The lateral velocity response amplitude of four-wheel steering mode is 67.23% of front wheel steering mode and the absolute offset is 29.58% of front wheel steering. Figure 7c shows the lateral displacement response curve. The curve indicates that the robot’s deviation from the 50 m circular radius. Negative deviation indicates that the robot is in a state of understeer and the actual turning radius is less than the set radius of 50 m. The growth rate of the lateral displacement of the front wheel steering mode is greater than that of the four-wheel steering mode. At the test termination time t = 30 s, the lateral displacement of the front wheel steering mode reaches −1.24 m. The lateral displacement of four-wheel steering mode is 0.42 m. The lateral deviation angle response curve is shown in Figure 7d. The amplitude of the lateral deviation angle of the four-wheel steering trackless auxiliary transport robot is 0.0018 rad, and the offset is −0.000619 rad. The amplitude of the front wheel steering lateral deviation angle is 0.002665 rad, and the offset is −0.00213 rad. The amplitude of the four-wheel steering mode lateral deviation angle is 67.54% of the amplitude of the front wheel steering mode lateral deviation angle and the absolute offset value is 29.06% of the front wheel steering mode. Figure 7e shows the response curve of the yaw rate. The amplitude of the yaw rate for four-wheel steering mode is 0.0064 rad/s, and the offset is 0.0996 rad/s. The amplitude of the yaw rate for front wheel steering is 0.0223 rad/s and the absolute offset is 0.11 rad/s. Figure 7f shows the driving trajectory of the trackless auxiliary transport robot. The average radius of the four-wheel steering trajectory is 50.12 m, with an average radius error of 2.4%. The radius of the front wheel steering mode trajectory is 45.63 m, with an average radius error of 8.74%. The four-wheel steering mode has a high degree of coincidence with the initial trajectory, and the front wheel steering mode robot deviates significantly from the initial trajectory, resulting in oversteering. At a speed of 40 km/h, the four-wheel steering mode trajectory tracking ability is better than the front wheel steering mode.

## 5 Conclusion

This study takes a trackless auxiliary transport robot with four-wheel steering and four-wheel drive capabilities as the research object, and constructs a four-wheel steering control strategy and a single input-output PID trajectory tracking control strategy with the walking speed of the trackless auxiliary transport robot as the system input. The following conclusions were drawn from testing the steering angle step, single lane manipulation stability, and circular path trajectory tracking ability of the wheeled transport robot in front wheel steering mode and four-wheel steering mode with a radius of 50 m.

(1) Under the low-speed 10 km/h step angle input test condition, compared to front wheel steering mode, the four-wheel steering system can reduce the turning radius by up to 32.2%, improving the passing ability of trackless auxiliary transport robot in narrow tunnel environments.
(2) The average radius error of the circular path tracking of the four-wheel steering trackless auxiliary transport robot at low speed of 10 km/h and high speed of 40 km/h is smaller than that of the front wheel steering mode. The trajectory tracking ability of the four-wheel steering mode for the planned path is better than the front wheel steering mode.

## Data Availability

The raw data supporting the conclusions of this article will be made available by the authors, without undue reservation.
